# Draft Genome Sequence of a Diarrheagenic *Morganella morganii* Isolate

**DOI:** 10.1128/genomeA.01165-15

**Published:** 2015-10-08

**Authors:** Pallavi Singh, Rebekah Mosci, James T. Rudrik, Shannon D. Manning

**Affiliations:** a Department of Microbiology and Molecular Genetics, Michigan State University, East Lansing, Michigan, USA; b Michigan Department of Health and Human Services, Lansing, Michigan, USA

## Abstract

This is a report of the whole-genome draft sequence of a diarrheagenic *Morganella morganii* isolate from a patient in Michigan, USA. This genome represents an important addition to the limited number of pathogenic *M. morganii* genomes available.

## GENOME ANNOUNCEMENT

*Morganella morganii* was originally identified as a cause of diarrheal disease (1) and is now recognized as an important opportunistic pathogen. It has been linked to catheter-based urinary tract infections, sepsis, and wound infections and has also been reported among immunocompromised, postoperative, and intensive care unit patients (2, 3). *M. morganii* was found to produce β-lactamases, which results in resistance to a set of clinically relevant antibiotics, while the *in vivo* transfer of an *M. morganii*-derived plasmid (*bla*_OXA-181_) encoding carbapenem resistance was recently observed in *Escherichia coli* cultured from a wound infection (4). To date, only a subset of *M. morganii* genomes have been sequenced (5–7); hence, additional genomes are needed to facilitate comparative genomic analyses and the identification of virulence and resistance genes.

The *M. morganii* isolate TW17014 was recovered in 2011 from the stool of an adult male who had been hospitalized after suffering from abdominal pains, diarrhea, and bloody diarrhea. Genomic DNA was extracted and purified using the Qiagen DNA extraction kit (Qiagen Sciences, MD). Whole-genome sequencing was performed with the Illumina MiSeq platform using 500 cycles with 250-bp paired-end reads following library preparation with the Nextera XT kit (Illumina, Inc., San Diego, CA). The draft genome (3,982,639 bp) with 32× coverage was assembled using Velvet 1.2.07 (8), and ambiguous sequences and adapters were trimmed using Trimmomatic (9), followed by quality checking using FastQC (http://www.bioinformatics.babraham.ac.uk/projects/fastqc/). The genome was annotated using the Prokaryotic Genomes Annotation Pipeline (10), which identified 3,873 genes with 3,724 coding sequences (CDSs), 9 rRNAs, 52 tRNAs, and 3 noncoding RNAs (ncRNAs). Functional annotation was carried out using the Rapid Annotations using Subsystems Technology (RAST) server (11), and 3,810 coding sequences with 59 RNAs were identified. Among the annotated subsystem features, 361 genes were identified as amino acids and derivatives, 307 genes were linked to carbohydrate metabolism, 299 genes encode cofactors or vitamins, and 237 genes were associated with protein metabolism. A total of 78 genes were found to be associated with virulence, disease, and defense, along with 21 phage-related genes. Use of the Resistance Gene Identifier (RGI) version 2 via the Comprehensive Antibiotic Resistance Database (12) identified 571 genes associated with antibiotic resistance, multidrug efflux transporter systems, macrolide efflux proteins, resistance-nodulation-cell division, and two-component regulatory systems. Antibiotic resistance genes targeting β-lactams (n = 2), chloramphenicol (n = 1), polymyxin (n = 4), lincosamide (n = 1), fosfomycin (n = 1) and mac/lin/phe/str/lin (n = 1) were found, as well as 16 genes associated with antibiotic efflux pumps. This *M. morganii* genome was most closely related to *Providencia rustigianii* DSM 4541 (score, 530), *Providencia alcalifaciens* DmeI2 (score, 306), and *Proteus mirabilis* WGLW4 and WGLW6 (scores, 280 and 272, respectively) genomes.

### Nucleotide sequence accession numbers.

The annotated draft genome has been deposited at DDBJ/EMBL/GenBank under the accession no. LFWB00000000. The version described in this paper is LFWB01000000.

## References

[1] Müller HE 1986 Occurrence and pathogenic role of *Morganella-Proteus-Providencia* group bacteria in human feces. J Clin Microbiol 23:404–405. 3517057 10.1128/jcm.23.2.404-405.1986 PMC268658

[2] O’Hara CM, Brenner FW, Miller JM 2000 Classification, identification, and clinical significance of *Proteus, Providencia*, *and Morganella*. Clin Microbiol Rev 13:534–546. doi:10.1128/CMR.13.4.534-546.2000. 11023955 PMC88947

[3] Lin TY, Chan MC, Yang YS, Lee Y, Yeh KM, Lin JC, Chang FY 2015 Clinical manifestations and prognostic factors of *Morganella morganii* bacteremia. Eur J Clin Microbiol Infect Dis 34:231–236. doi:10.1007/s10096-014-2222-8. 25107625

[4] McGann P, Snesrud E, Ong AC, Appalla L, Koren M, Kwak YI, Waterman PE, Lesho EP 2015 War wound treatment complications due to transfer of an IncN plasmid harboring *bla*(OXA-181) from *Morganella morganii* to CTX-M-27-producing sequence type 131 *Escherichia coli*. Antimicrob Agents Chemother 59:3556–3562. doi:10.1128/AAC.04442-14. 25870058 PMC4432134

[5] Chen YT, Peng HL, Shia WC, Hsu FR, Ken CF, Tsao YM, Chen CH, Liu CE, Hsieh MF, Chen HC, Tang CY, Ku TH 2012 Whole-genome sequencing and identification of *Morganella morganii* KT pathogenicity-related genes. BMC Genomics 13(Suppl 7):S4. doi:10.1186/1471-2164-13-S7-S4. PMC3521468 23282187

[6] Khatri I, Dureja C, Raychaudhuri S, Subramanian S 2013 Draft genome sequence of the opportunistic human pathogen *Morganella morganii* SC01. Genome Announc 1(1):e00051-12. doi:10.1128/genomeA.00051-12. 23405333 PMC3569328

[7] Nash JH, Young NM 2015 Draft whole-genome sequence of *Morganella morganii* serotype O:1ab. Genome Announc 3(3):00453-15. doi:10.1128/genomeA.00453-15. PMC4440951 25999571

[8] Zerbino DR, Birney E 2008 Velvet: algorithms for *de novo* short read assembly using de Bruijn graphs. Genome Res 18:821–829. doi:10.1101/gr.074492.107. 18349386 PMC2336801

[9] Bolger AM, Lohse M, Usadel B 2014 Trimmomatic: a flexible trimmer for Illumina sequence data. Bioinformatics 30:2114–2120. doi:10.1093/bioinformatics/btu170. 24695404 PMC4103590

[10] Angiuoli SV, Gussman A, Klimke W, Cochrane G, Field D, Garrity G, Kodira CD, Kyrpides N, Madupu R, Markowitz V, Tatusova T, Thomson N, White O 2008 Toward an online repository of Standard Operating Procedures (SOPs) for (meta)genomic annotation. Omics 12:137–141. doi:10.1089/omi.2008.0017. 18416670 PMC3196215

[11] Aziz RK, Bartels D, Best AA, DeJongh M, Disz T, Edwards RA, Formsma K, Gerdes S, Glass EM, Kubal M, Meyer F, Olsen GJ, Olson R, Osterman AL, Overbeek RA, McNeil LK, Paarmann D, Paczian T, Parrello B, Pusch GD, Reich C, Stevens R, Vassieva O, Vonstein V, Wilke A, Zagnitko O 2008 The RAST server: Rapid Annotations using Subsystems Technology. BMC Genomics 9:75. doi:10.1186/1471-2164-9-75. 18261238 PMC2265698

[12] McArthur AG, Waglechner N, Nizam F, Yan A, Azad MA, Baylay AJ, Bhullar K, Canova MJ, De Pascale G, Ejim L, Kalan L, King AM, Koteva K, Morar M, Mulvey MR, O’Brien JS, Pawlowski AC, Piddock LJ, Spanogiannopoulos P, Sutherland AD, Tang I, Taylor PL, Thaker M, Wang W, Yan M, Yu T, Wright GD 2013 The comprehensive antibiotic resistance database. Antimicrob Agents Chemother 57:3348–3357. doi:10.1128/AAC.00419-13. 23650175 PMC3697360

